# Nondestructive Testing Based Compressive Bearing Capacity Prediction Method for Damaged Wood Components of Ancient Timber Buildings

**DOI:** 10.3390/ma14195512

**Published:** 2021-09-23

**Authors:** Lihong Chang, Wei Qian, Hao Chang, Xiaohong Chang, Taoping Ye

**Affiliations:** 1 Department of Rural Regional Development, College of Humanities and Urban-Rural Development, Beijing University of Agriculture, Beijing 102206, China; changlhong@126.com; 2 Department of Urban Planning, College of Architecture and Urban Planning, Beijing University of Technology, Beijing 100124, China; 3 Beijing Engineering Research Center of Historic Buildings Protection, Beijing 100124, China; 4 Department of Mechanical and Electronic Engineering, School of Mechanical and Precision Instrument Engineering, Xi’an University of Technology, Xi’an 710048, China; 5 Department of Financial Management, School of Accountancy, Beijing Wui Zi University, Beijing 101149, China; changhlw@126.com; 6 The First Construction Engineering Company Ltd., China Construction Second Engineering Bureau, Beijing 100176, China; taskaction@163.com

**Keywords:** wood components, internal damage, nondestructive testing, compressive strength

## Abstract

In this research, a wave-drag modulus nondestructive testing method was proposed to predict the compressive bearing capacity of damaged wood components. Using an ancient Chinese building as a case study, internal and external inspections were performed to obtain defect data and related tree species information. Using the same tree species, wave-drag modulus and scale tests were carried out to predict the residual bearing capacity when there was damage in the form of internal cavities or edge material reduction and to compare the damage and loss experimental data. The results show that the internal defect combination model established by two nondestructive testing methods (stress wave and impedance meter) based on the weight distribution can accurately determine the internal damage condition of wood components. There was a significant correlation between wave-drag modulus and compressive strength along the wood grains. The measured values of wood components with different defects were consistent with the theoretical values predicted by the wave-drag modulus, which can effectively improve the prediction of residual bearing capacity. In addition, it was determined that edge material reduction is more destructive to a wood component than the presence of an interior cavity. Thus, the wave-drag modulus can quickly locate vulnerable sections and provide a relevant basis for judging the material condition of wood components in ancient buildings.

## 1. Introduction

Ancient buildings constructed of wood are of high historical, scientific, and artistic value. The wood in most ancient Chinese buildings is used as load-bearing structures. However, wood is a biological material. Therefore, owing to the influence of temperature, humidity, and service life, the wood components will suffer from different types and degrees of damage [1,2]. Degradation seriously affects the mechanical properties of wood components [3] and can lead to local damage or overall collapse. Consequently, this would cause the irreparable loss of these nonrenewable heritage buildings [4]. Research examining the material properties of wood components in ancient buildings is of great significance because it can be used to predict the residual bearing capacity of these components. In order to protect the authenticity of ancient buildings, methods such as background analysis, naked-eye observation, hammer percussion, monitoring and evaluation, and moisture content detection are often used to identify the residual bearing capacity of wood components [5]. These methods are simple but are heavily influenced by experience due to the lack of quantitative data support. Some scholars disassemble wood components to test their physical and mechanical properties in the laboratory.

Although the experimental data collected in this way are more accurate, the wood components are seriously damaged during testing and can no longer be utilized, which can seriously damage the original nature of ancient buildings. Nondestructive testing has attracted considerable research attention due to the minimal damage inflicted on wood components [6,7,8,9]. Research on the nondestructive testing of wood components has been carried out across the world [10]. The Pilodyn detection method is a nondestructive testing instrument that first appeared in Sweden and was especially used for the safety inspection of electric poles, which has been widely used in ancient buildings. Testing of timber structure and ancient wood protection [11]. Through the Pilodyn study, a nondestructive testing technique, a study of the density and fiber length of pine, it was found that the Pilodyn value has a significant correlation with the density, which implies that it can be used to predict the density in the radial direction [12,13]. The relationship between stress wave velocity and dynamic elastic modulus of *Pinus palustris* was established by Zhu [14] through studying the dynamic elastic modulus of *Pinus palustris* as predicted by stress waves. In addition, Wang et al. [15] carried out a study on impedance curves that found that impedance testing technology can accurately identify decayed and transitional areas inside the component, which provides a new strategy for making material predictions. Using a combination of stress wave and impedance meter (also known as resistograph) technology, Zhang et al. [16] established an internal defect simulation model and identified partial internal defects. This method could be helpful to determine the material properties of wood components in the future. Through comprehensive consideration of the convenience and ease of field application [17], the stress wave and impedance meter were selected as the main objects of this research. At present, this method is widely used in trees, but the structure, load, and moisture content of the wood components of ancient buildings are unique. The methods applied to living wood cannot be directly applied to the wood components of ancient buildings, which makes it difficult to predict the bearing capacity of the wood components. Therefore, because of the complexity of wood components in ancient buildings and to ensure their authenticity, it is necessary and urgent to predict the residual bearing capacity of wood components using nondestructive testing methods.

This paper proposes a new formal method, a nondestructive testing method that was used to collect data from wood components in ancient buildings. The data were matched with that collected from the same tree species to perform the experiment. The relationship between compressive strength and wave-drag modulus was studied in small specimens. In addition, referring to Building Methods [18,19] and other literature, 10 wooden column specimens of reduced scale were designed, and the damage type and location were simulated on-site. By comparing the data collected via nondestructive testing and the physical pressure testing machine, the residual bearing capacity and vulnerable parts of the wood members were predicted, which provides a reference for the evaluation of wood component diseases in ancient buildings and provides data support for the repair and reinforcement of wood components in the future.

## 2. Ancient Building Case Study

**Figure 1:**
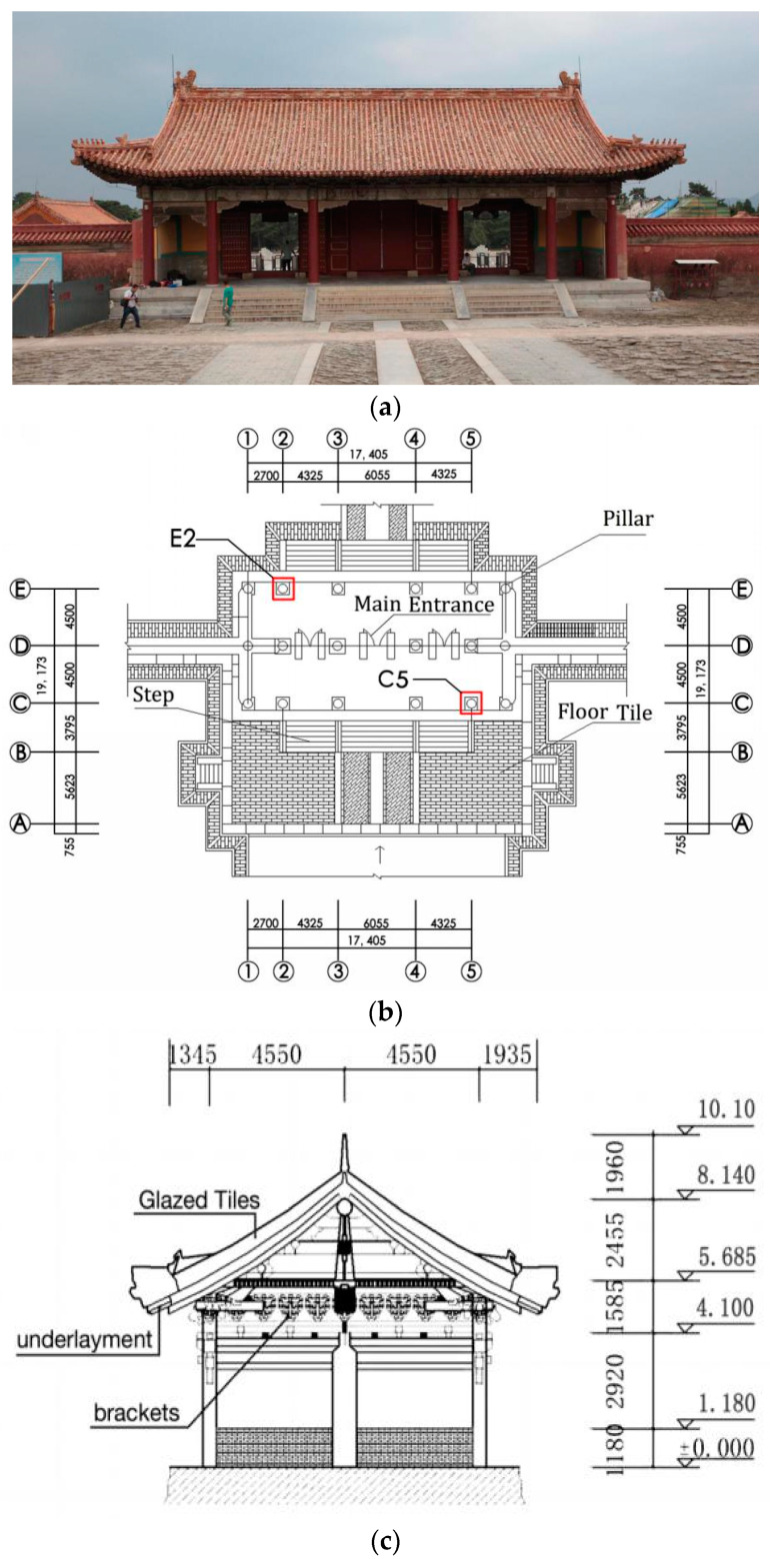
Ancient building: (**a**) Scene; (**b**) Floor plan of ancient building; (**c**) Section of ancient building.

The case study building (Gongmen) is located in Hebei Province, China. It was built during the Ming and Qing dynasties. Some of the wood components were repaired in 1954; however, records on the repair and protection of the components during that time are lacking (Figure 1). As the service life increases, the wood components continue to age and damage can be visually observed. Specific tests were conducted as explained in the following sections.

### 2.1. Surface Damage

**Figure 2:**
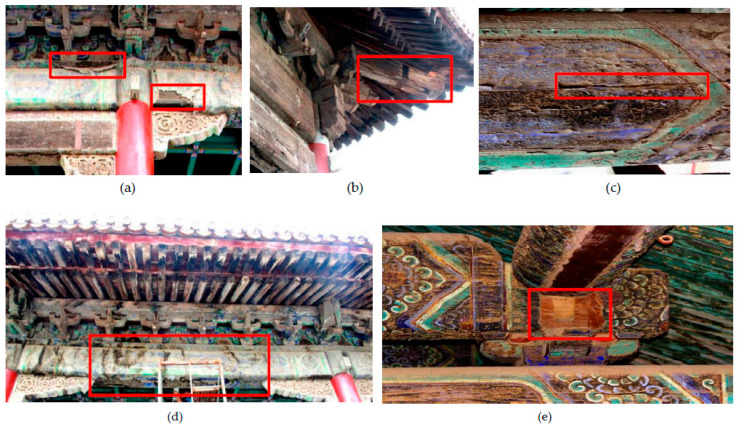
Surface damage to the building: (**a**) Wall shedding; (**b**) Mold in rafters; (**c**) Beam splitting; (**d**) Kick-up of frame; (**e**) Missing components.

Due to changes in temperature and humidity that occur in an outdoor climate, there is a difference in expansion coefficients between the colored painted layer [20,21], ground layer, and base layer of the wood components. Repeated expansion cycles lead to weak adhesion between layers, resulting in local separation from the base layer (Figure 2a). As a result of rainwater erosion and temperature changes, mold and the partial splitting of wood components have appeared on the wood rafters in the upper part of the buildings (Figure 2b,c). The wood material is loose due to internal and external damage to the wood components. Long-term loading of the wooden frame has led to bending and fracture of the joints, and components being missing from the structure (Figure 2d,e).

### 2.2. Internal Damage Detection

**Figure 3:**
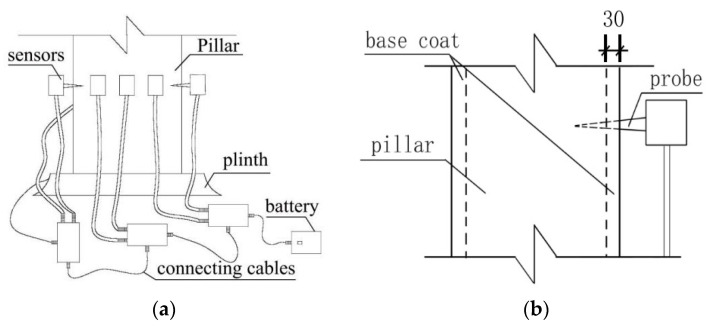
Schematic layout of the wood component testing process using the stress wave sensor: (**a**) Layout of stress wave sensors; (**b**) Penetration depth of stress wave sensor.

The moisture content meter (Probe type, MT-10/MT-15 model, the manufacturer of the equipment is Shenzhen Honglong instrument Co., Ltd., and the place of production comes from Shenzhen, China) is used to sample and inspect the two ends and the middle of the load-bearing structure of wooden pillars and beams. It should be noted that the number of inspections for each component shall not be less than 6. The moisture content value is collected and the average value is calculated to be 12%. After determining the height of the measured section, using a Fakopp stress wave sensor (The manufacturer of this equipment is Hungary Fakopp company, and the place of production comes from Agfalva, Hungary) and 10 probes, the average distribution of the cross-section of the wood column was determined (Figure 3a). To avoid errors in the internal damage testing results caused by the ground layer outside the column, the probe depth of the stress wave sensor into the column was set to more than 30 mm (Figure 3b). According to the damage position shown in the stress wave detection diagram, the impedance meter can selectively select the detection path direction, so that the detection path can pass through the damaged part more effectively. Nondestructive testing of the wood components was carried out to determine the internal residual area. In addition to detecting the damage of the horizontal section of the wooden column, the observation also involves whether the wooden column still has extensible damage in the longitudinal direction. Therefore, under the test background of 6 cross-sections with different heights, the distance from the bottom of the column is 10 cm, 20 cm, 50 cm, 100 cm, 120 cm, 140 cm (from large internal damage to no damage found). With Arbor Sonic 3D software (5.2.107 version, 2015. The manufacturer of the software is Hungary Fakopp company, and the place of production comes from Agfalva, Hungary.), the 3D distribution model of the wooden column (100–1400 mm above the column foundation) is established.

**Figure 4:**
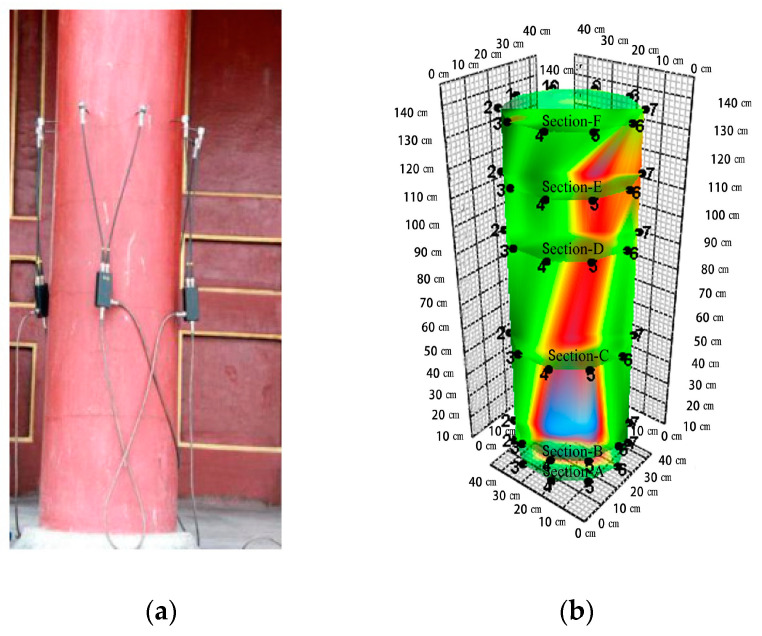
Nondestructive testing of stress wave: (**a**) Stress wave data acquisition; (**b**) Internal damage imaging model. Note: Green represents healthy material, red represents loose material with damage, and blue represents interior cavities.

The results show that C5 and E2 had serious internal damage (Figure 1), especially C5. The wooden column was damaged from the middle (100–1400 mm above the column foundation) to the internal edge (Figure 4).

The general position of the damage can be estimated using the stress wave model of internal damage and analyzing the transmission velocity between stress wave sensors.

**Figure 5:**
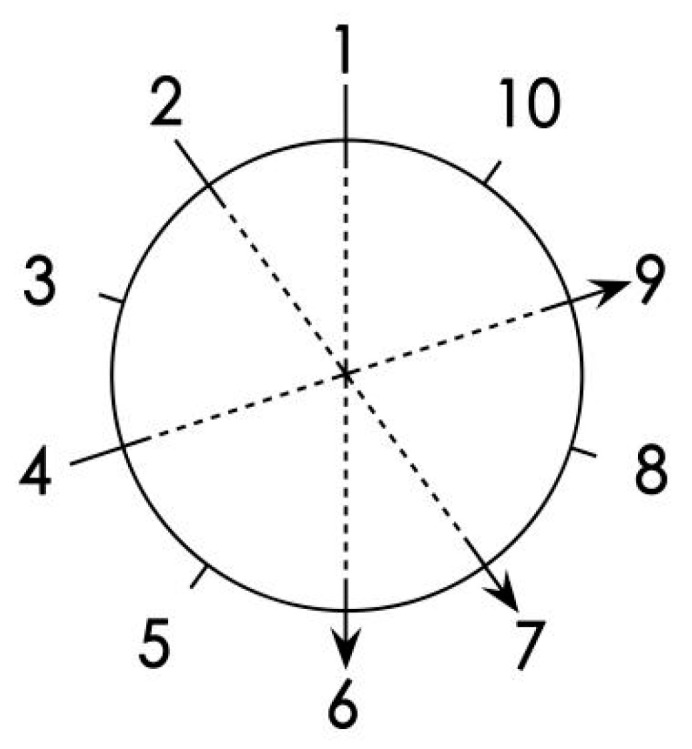
Internal testing path of the impedance meter.

Detection using an impedance meter (IML-RESI PD 500 model, the manufacturer of the equipment is IML Instrumenta Mechanik Labor System GmbH, and the place of production comes from Wiesloch, Germany.). Three testing paths (lateral, vertical, and chordal, different angles for maximum coverage of the test area: stress sensor locations 1–6, 2–7, and 4–9) can be selected as the internal testing path of the impedance meter within the wood column (Figure 5). The targeted stress wave damage area can be corrected by the impedance meter.

**Figure 6:**
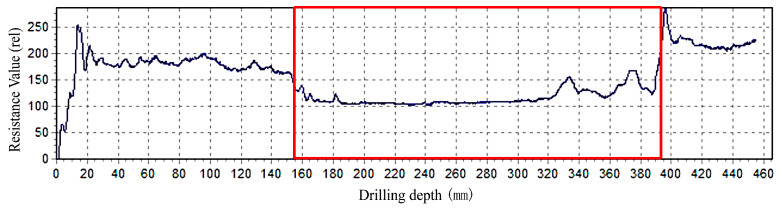
Example of the impedance of internal defects in column C5 Note: 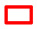 Represents the position and length of defects in the path under relative resistance.

The impedance meter results show that the relative resistance value in the middle of the wood column was obviously lower than that of the surrounding material; this difference can be used to clearly distinguish the specific boundary of the internal damage. Take the distance (200 mm) from wood column C5 to the column foundation as an example (Figure 6).

Suppose the testing section is T, the damage testing value of stress wave *S*_1_ is 884.1 cm^2^, and the damage testing value of the impedance meter *S*_2_ is 743.8 cm^2^. Different internal nondestructive testing methods will produce the total error of the combined prediction. The total error uses the Shapley value for weight distribution to obtain the weight of each prediction method in the combined prediction. Then, the Shapley value coupling method can be used to calculate the internal damage of the testing section T [22,23]. The gross error of the combined prediction model will be:

(1) $$\varepsilon =(884.1+743.8)÷2=813.9{\;\mathrm{cm}}^{2}$$

Based on the relevant concept of the Shapley value, the gross error of the combined prediction model is *I* = {1,2}, and the error of each subset is *ε* {1}, *ε* {2}, and *ε* {1,2}. Applied to section T, the mean absolute value of the subset error is 884.1 cm^2^, 743.8 cm^2^, and 813.9 cm^2^.

According to the weight distribution formula of the Shapley value:

(2) $$\varepsilon_{i}^{\prime }=\sum_{d_{i}\in d}w(\left|d\right|)\left[\varepsilon \left(d-\left\{i\right\}\right)\right]$$

(3) $$w\left|d\right|=\frac{\left(n-\left|d\right|\right)!\left(\left|d\right|-1\right)!}{n!}$$

where *d*−{*i*} is the removal model in the combined prediction *i*; *i* is the certain nondestructive testing prediction model in the combined prediction model; *ε*′*_i_* is the error contribution in the combined prediction model *i*, i.e., the Shapley value; *w*|*p*| is a weighting factor, representing the combined marginal contribution to the combined prediction of nondestructive testing *i*; *d* is any subset of *I*; |*d*| is the quantity of combined prediction models used in the nondestructive testing.

According to Equations (2) and (3), the calculation formula of the weight of each prediction method in the nondestructive testing can be obtained:

(4) $$w_{i}=\frac{1}{n-1}\times \frac{\varepsilon -\varepsilon_{i}}{\varepsilon },i=1,2,\cdots ,n$$

Accordingly, the contribution error *Ɛ*_1_ of stress wave nondestructive testing of section T is:

(5) $$\varepsilon_{1}=\frac{1!\times 0!}{2!}\left[\varepsilon \left\{1\right\}-\varepsilon \left(\left\{1\right\}-\left\{1\right\}\right)\right]+\frac{0!\times 1!}{2!}\left[\varepsilon \left\{1,2\right\}-\varepsilon \left\{1,2\right\}-\varepsilon \left\{1\right\}\right]=477.1{\;\mathrm{cm}}^{2}$$

Similarly, the error *ε*_2_ = 336.8 cm^2^ of the impedance meter of the cross-section can be obtained. It is verified that: *ε*_1_ + *ε*_2_ = 813.9 cm^2^.

This indicates that the sum of contribution errors from the two nondestructive testing methods (stress wave and impedance meter) in section T is equal to the gross error of the combined prediction *ε*. The contribution value also reflects the accuracy of each nondestructive testing method.

The distribution weight of stress wave nondestructive testing with Equation (4) can be obtained using: $w_{1}=0.41$.

In the same way, the distribution weight of impedance meter nondestructive testing is $w_{2}=0.59$.

The combined model of internal defects in section T of wood column C5 is $f=w_{1}\times s_{1}+w_{2}\times s_{2}$.

The internal damage type of the wood column was the middle cavity, and the damaged area was 801.32 cm^2^. In order to determine the bearing capacity of wood components, it is necessary to establish and analyze the relationship between nondestructive testing and material properties.

## 3. Reverse Experimental Detection

The compressive strength was tested in small specimens of wood components along the grains; a correlation was established in terms of density and wave-drag (stress wave and impedance). Based on the common wood column damage, a wood column specimen scale was made. The residual bearing capacity was predicted by wave-drag nondestructive testing, and these results were compared with those of the physical and mechanical experiments.

### 3.1. Nanocarbon-Based Electrodes

#### 3.1.1. Small Specimen Material

In order to reduce the errors caused by different tree species, timber components were tested by tree species sampling. A number of representative growth rings were selected and cut into small wooden blocks, 1.5 cm along the grain direction, in tangential, and radial directions. The resulting slices were observed by optical microscopy (The manufacturer of the equipment is Nikon Corporation, and the place of production comes form Tokyo, Japan.), to investigate the structural features of the three sections in the vertical, horizontal, and tangential directions. After identification, the tree species was determined to be pinewood. Therefore, among all possible tree species, mature hardwood pine was selected as the test tree species.

**Figure 7:**
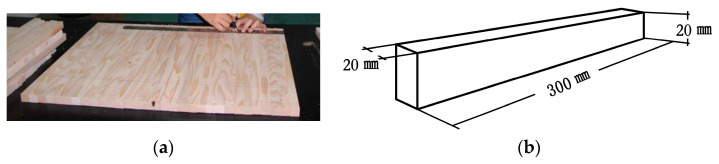
Small specimen: (**a**) Draw lines; (**b**) Small specimen size.

According to GB/T 1936.1-2009 [24] and GB/T 1935-2009 [25] related specimen testing standards, hardwood pine was sawn into small specimens of dimensions 20 mm × 20 mm × 300 mm (Figure 7). Specimens with branch knots and cracks were eliminated. The final specimens were numbered separately; there were a total of 131 small specimens. Specimens were maintained in a constant temperature and humidity chamber (The manufacturer of this equipment is Harbin Donglian Electronic Technology Development Co., Ltd., and the place of production comes from Harbin, China). The experiment used the MT-10/MT-15 model (probe type) moisture content measuring instrument. When the moisture content reached 12%, it was taken out for testing.

#### 3.1.2. Scaled Specimen Materials

**Figure 8:**
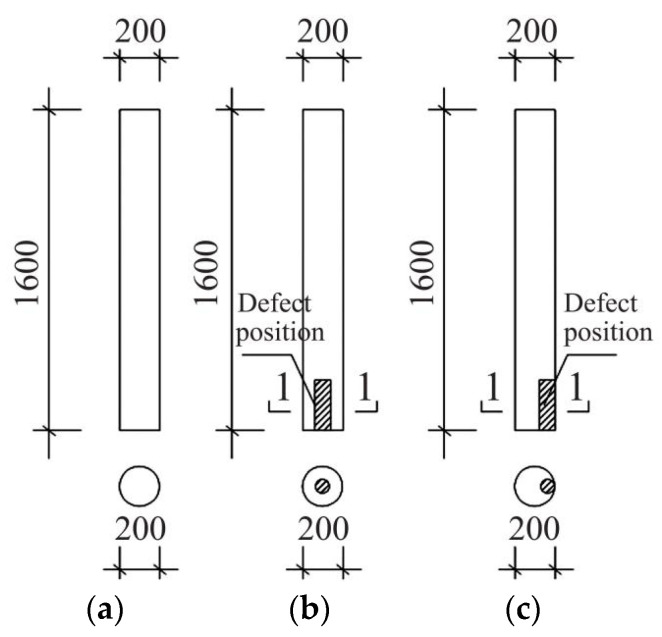
Size and position of damage to scaled specimens: (**a**) Undamaged; (**b**) Core material cavity; (**c**) Edge damage.

According to the methods of constructing and processing building structures described in Building Methods, 10 wood column scale specimens were made at a depth-width ratio of 1:8. They were divided into groups labeled A, B, and C. Group A included healthy specimens. Based on the site inspection of ancient buildings, most of the damage involved internal voids and weakened edge materials. Therefore, the specimens simulated the site damage types that were most commonly observed. Group B included four internal hollow specimens of different sizes and Group C included four edge reduction specimens of different sizes. For a list of the primary parameters, refer to Table 1. The structure and damaged parts of the specimen are shown in Figure 8. The specimens were kept in the laboratory (temperature 20 ± 5 °C, relative humidity 65 ± 5%) for three months until the moisture content reached 12% [26].

### 3.2. Experimental Methods

#### 3.2.1. Data Acquisition Path and Small Specimen Loading Test

A Hungarian FAKOPP two-probe stress wave tester (The manufacturer of the equipment is Hungary Fakopp company, and the place of production comes from Agfalva, Hungary.) was used to measure the propagation velocity of stress waves. Measuring points were selected along the longitudinal direction of the specimens. The distance between measuring points was measured. The two stress wave sensors were inserted into the specimen at an angle of 45° at the designated measuring points [27]. The propagation time was recorded by measuring the time of impact of the hammer on the sensor. In the experiment, the first tap reading was invalid and the time was counted from the second tap.

Three consecutive taps were applied, and three stress wave propagation times were obtained. The average time was used as the time determination result.

A German IML impedance meter (The manufacturer of the equipment is IML Instrumenta Mechanik Labor System GmbH, and the place of production comes from Wiesloch, Germany.) was used to drill micro drill needles (1.5 mm) into specimens at a constant rate, and a relative resistance was produced inside the specimens. The magnitude of the resistance reflects changes in density. The impedance meter inputs the resistance parameters generated during the detection process into the computer through the microcomputer system to display the resistance curve image and the resistance data of each distance in the detection path. The average value of the resistance data is determined as the resistance value of the specimen under a single detection path. In order to ensure the accuracy of the test piece data, both ends of each test piece are data collected to form two sets of resistance values *F*_1_ and *F*_2_. The overall average resistance of the small specimens was the average of these two values: *F*_S_ = (*F*_1_ + *F*_2_)/2.

**Figure 9:**
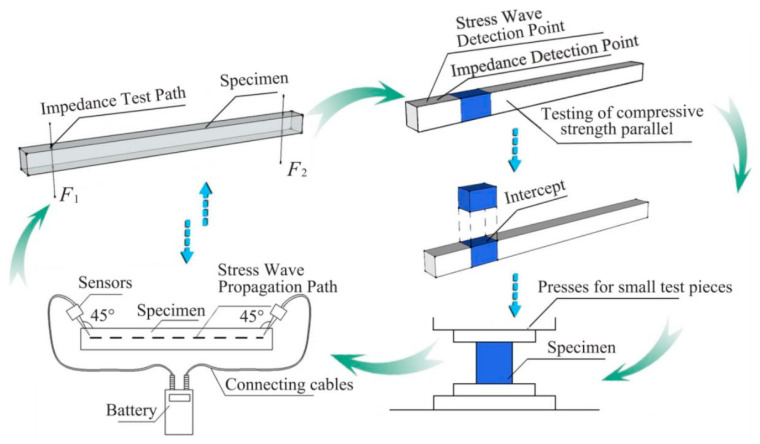
Small specimen test data acquisition flowchart.

After the small specimens were tested using stress waves and the impedance meter, sections of 20 mm × 20 mm × 30 mm were removed from the undamaged portion for density determination and to perform compression experiments along the grains. Compression experiments were performed using a universal mechanics testing machine operating at a rate of 3 mm/min to obtain the compressive strength σ and establish the relationship between the nondestructive testing parameters and the physical and mechanical properties of wood materials (Figure 9).

#### 3.2.2. Scaled Specimen Collection Paths and Loading Test

Considering that the scaled timber column specimens were circular, they were equally divided into three intervals (A, B, and C), and the stress wave velocity collection was performed separately on each section to obtain accurate data. The three sections (A, B, C) of the scaled specimen formed three stress wave data collection paths (*T_a_*, *T_b_*, *T_c_*). To ensure accuracy, each path was tested three times. Therefore, a total of nine detection paths were formed. The average value of the propagation time of the nine groups of stress waves was used as the measurement result of the stress wave propagation of the specimen, that is, the *T* (m·s^−1^) value. *T* was calculated as follows:

(6) $$T=\frac{T_{a1}+T_{a2}+T_{a3}+T_{b1}+T_{b2}+T_{b3}+T_{c1}+T_{c2}+T_{c3}}{9}$$

**Figure 10:**
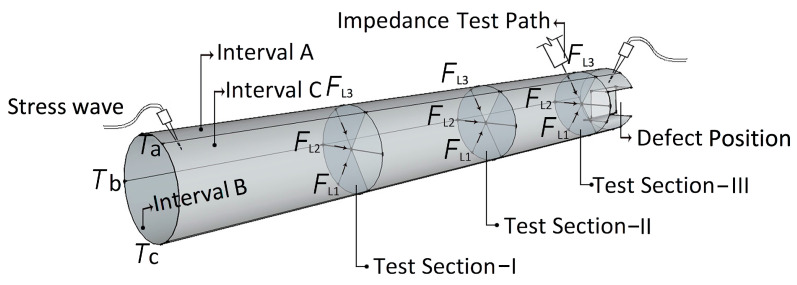
Scaled specimens test data acquisition flowchart.

Because the ends of the scaled timber column specimen were damaged, the impedance value includes the two ends and the middle, which together form three sections (Section-I, Section-II, and Section-III), and three paths were collected per section (*F*_L1_, *F*_L2_, *F*_L3_). The average value of each section was taken as the resistance value of the section. The overall average impedance of the scaled timber column specimen was: *F*_L_ = (Section-I + Section-II + Section-III)/3 (Figure 10).

**Figure 11:**
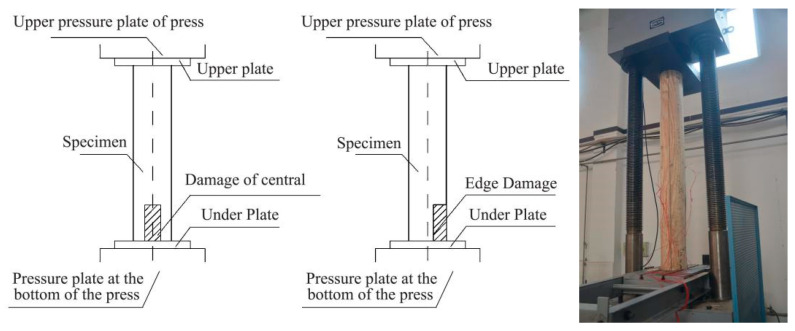
Scaled specimens test.

According to Equation (6), the data of the stress wave and the impedance meter were collected and formed the wave-drag modulus (the propagation velocity of the stress wave and the resistance value of the impedance meter). After collecting the wave-drag data using nondestructive testing methods, a microcomputer-controlled electro-hydraulic servo compression testing machine (YAW-3000 A, the manufacturer of this equipment is Jinan Shijin group Co., Ltd., and the place of production comes from Jinan, China.) was utilized to apply a load to the scaled specimens. The loading rate was adjusted to 5 mm/min, and specimens that had undergone stress wave and impedance meter testing were placed in the compression testing machine so that load was applied along the grain direction. When the horizontal load of the specimen dropped below 85% of the peak load, the appearance of the specimen was observed. When significant damage occurred, the loading was stopped. The obtained compressive strength *σ*_1_ of the timber column specimen can be used to verify the relationship between the wave-drag prediction data collected using nondestructive testing and the mechanical properties determined by physical testing (Figure 11).

## 4. Numerical Analysis and Prediction Model

### 4.1. Analysis of the Correlation between Single Nondestructive Testing Value and Compressive Strength

When the probe entered the wood when the impedance meter was testing, the rotation speed value *F_drill_* and the forward speed value *F_feed_* was generated. 131 small specimens were tested to calculate average rotation speed $\bar{F_{drill}}=23.25$, and average forward speed $\bar{F_{feed}}=64.48$. In order to measure the correlation between physical and mechanical properties and the results of nondestructive testing, after nondestructive testing, each small specimen was cut into 30 mm × 20 mm × 20 mm sections for compressive strength testing along the grain. Assuming that the strength along the grain direction is *σ* and the average stress wave velocity measured using nondestructive testing is *V*, $\bar{V}=4.641$ mm/µs. Through the 131 small specimens one by one along with the grain compressive strength test, the average value of the data obtained was $\bar{\sigma }=66.51$ MPa.

**Figure 12:**
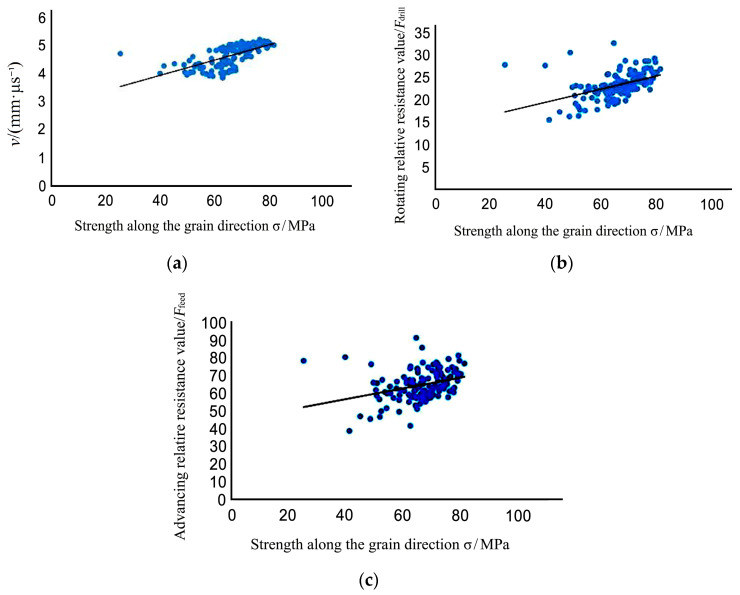
Relationship between nondestructive testing and compressive strength of small specimens: (**a**) Compressive strength and stress wave velocity; (**b**) Compressive strength and rotating relative resistance value; (**c**) Compressive strength and advancing relative resistance value.

As shown in Figure 12, there is a linear relationship between wave velocity, resistance value, and tensile strength parallel to the grain:

*σ* = 0.0274*v* + 2.8265 (*r*^2^ = 0.4282) (7)

*σ* = 0.1464 *F_drill_* + 13.591 (*r*^2^ = 0.1066) (8)

*σ* = 0.3051 *F_feed_* + 44.35 (*r*^2^ = 0.199) (9)

σ is the specimen compressive strength, *v* is the specimen stress wave velocity, *F_drill_* is the rotating relative resistance value, and *F_feed_* is the advancing relative resistance value.

Figure 10 indicates that there is a positive correlation between small specimen wave velocity, relative resistance value, and compressive strength. The correlation between stress wave velocity, relative resistance value, and compressive strength is relatively low. It can be concluded that the use of a single value stress wave velocity value, rotating relative resistance value, or needle-inclined relative resistance value for predicting compressive strength has certain shortcomings.

### 4.2. Correlation between Density and Nondestructive Testing Value 

The density of a specimen can reflect the propagation velocity of stress waves, and this can be used to determine the dynamic elastic modulus as follows:

(10) $$E=pv^{2}$$

where *E* is the specimen dynamic elastic modulus, *p* is the specimen air-dried density, and *v* is the propagation velocity of the stress wave.

As wood is a nonuniform material, density is one of the important parameters in the elastic modulus. In order to explore the relationship between wave velocity *v* and resistance values (*F_drill_* and *F_feed_*), air-dried analysis was performed on some sections of 131 small specimens.

Taking density as the independent variable *p* and resistance value as the dependent variable *F*, the following fitting equation was obtained:

*p* = 0.0109 *F_drill_* + 0.4508 (*r*^2^ = 0.4988), (11)

*p* = 0.0039 *F_feed_* + 0.455 (*r*^2^ = 0.5085), (12)

*p* = −0.01*v* + 0.756 (*r*^2^ = 0.0083), (13)

**Figure 13:**
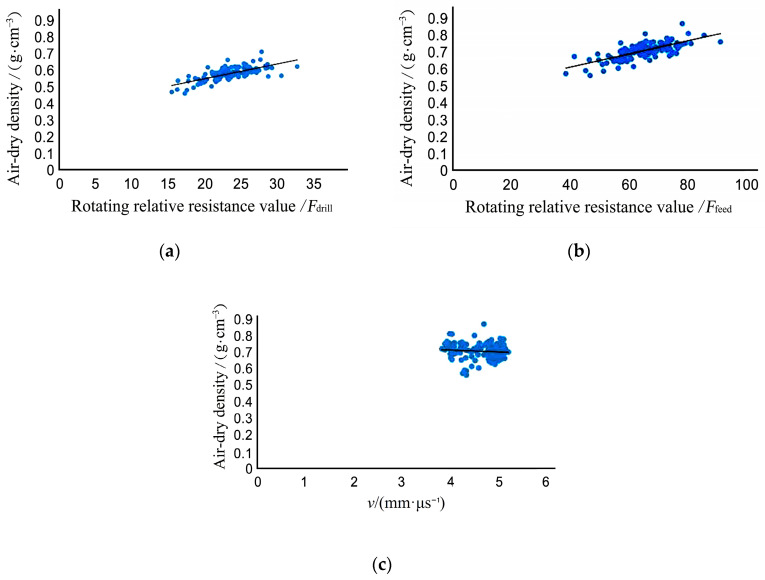
Relationship between density and nondestructive testing of small specimens: (**a**) Resistance values (*F_drill_*) and density; (**b**) Resistance values (*F_feed_*) and density; (**c**) Wave velocity and density.

As shown in Figure 13, the correlation between wave velocity and density is relatively poor. However, the impedance value *F* can more accurately reflect the internal density of the wood. The coefficient *r*^2^ of *F_drill_* and *F_feed_* is basically the same, and both reflect the material distribution inside the wood. However, the visual expression of the forward speed value in the impedance meter software analysis is more intuitive and easier to understand. In other words, *F_feed_* can more accurately reflect the density distribution within the wood’s interior.

According to Equations (10) and (12), *v*^2^ and *F_feed_* can be used to predict the mechanical properties of wood components; *F_feed_ v*^2^ is defined as the wave-drag modulus.

### 4.3. Correlation between Wave-Drag Modulus and Compressive Strength

Using the propagation speed of the stress wave and the relative resistance value of the impedance meter as independent variables, the *σ* of the small specimens could be analyzed. In order to avoid risking the safety of a timber component if the mechanical properties predicted by nondestructive testing are higher than the actual values, a regression line with 95% reliability was used to estimate the physical and mechanical property values of the wood. The resulting correlation is:

*σ* = 1.9113 *F_feed_ v*^2^ + 31.5078 (*r*^2^ = 0.635) (14)

**Figure 14:**
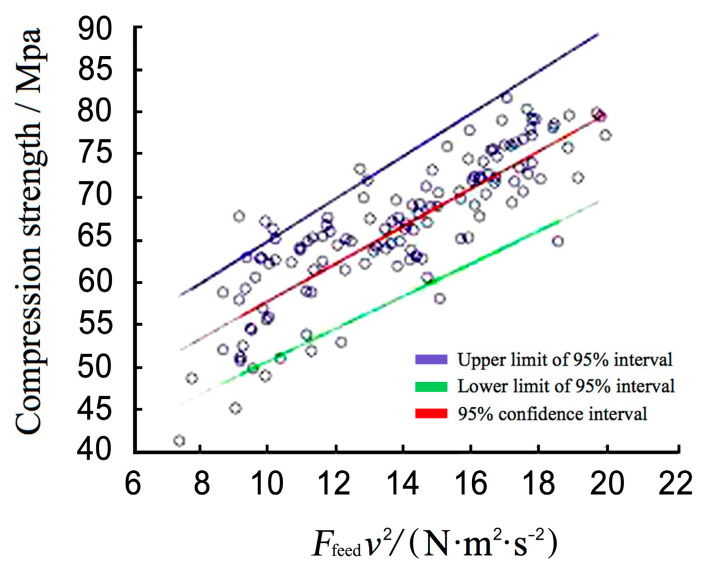
Correlation between wave-drag modulus and compressive strength.

As shown in Figure 14, utilizing the wave-drag modulus to predict the mechanical properties of small specimens provides more accurate results than using a single type of nondestructive testing.

### 4.4. Prediction of Compressive Bearing Capacity Modulus

The compressive bearing capacity of the scale specimen can be predicted using the wave-resistance modulus of a small specimen. This was demonstrated using cross section-I of the reduced scale specimen as an example.

There are three straight lines in the testing path of the reduced scale specimen impedance meter. Suppose the testing paths are TL_1_, TL_2_, and TL_3_. The three testing paths are in the same horizontal plane, which is the testing section of the reduced scale specimen. TL_1_, TL_2_, and TL_3_ intersect at the center of the testing section of the reduced scale specimen, and the included angle is 60°.

TL1, TL2, and TL3 divide the testing section into six areas, i.e., areas *a*, *b*, *c*, *d*, *e*, and *f*. Therefore, the testing area of the reduced scale specimen is *S*, i.e.,

(15) $$S=S_{a}+S_{b}+S_{c}+S_{d}+S_{e}+S_{f}$$

Taking area a as an example, the testing path on both sides of area a is divided into equal distance segments of 5 mm from the edge to the center. Area a can be divided into several parts (*S_a_*_1_, *S_a_*_2_, …, *S_ax_*, … *S_an_*) by connecting the dividing points on both sides. As a result:

(16) $$S_{a}=\sum_{i=1}^{n}S_{ax},(1\leq x\leq n,x\in N^{+})$$

**Figure 15:**
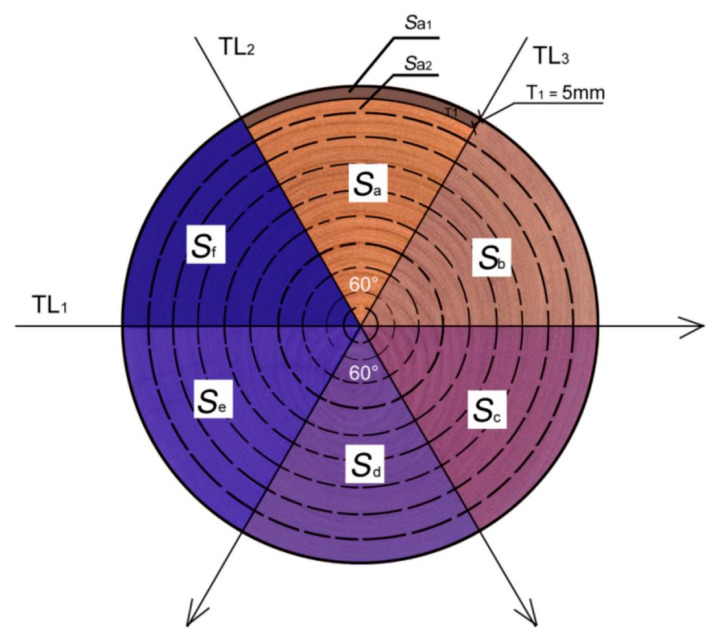
Strength schematic diagram of test section division.

A partition diagram is shown in Figure 15. If the radius of each specimen testing section is *r*, can be obtained according to the sector area formula, then:

(17) $$S_{ax}=\frac{\pi {\left[r-5\left(x-1\right)\right]}^{2}}{6}-\frac{\pi {\left(r-5x\right)}^{2}}{6}=\frac{5\pi \left(2r-10x+5\right)}{6},x\in N_{+}$$

The other areas can be calculated in the same way.

The path forms a closed interval every 5 mm. In the case of the *S_a_*_1_ area, the average resistance *F_i_* and stress wave velocity corresponding to the scaled specimen *S_a_*_1_ interval can be obtained along the detection path. According to Equation (14), which models the relationship between the compressive strength obtained by mechanical testing of the small specimen and the measured nondestructive testing parameters, the corresponding compressive strength can be calculated as:

(18) $$e_{a1}=1.9113F_{i}v^{2}+31.5078$$

Assuming that the residual compressive capacity of the specimen detection section is *N*, then the residual compressive capacity of *S_ax_* is *N_ax_* (*x* = 1, 2, 3, ..., *n*), and the compressive capacity of *S_a_*_1_ is *N_a_*_1_.

(19) $$N_{a1}=e_{a1}\times S_{a}{}_{1}$$

(20) $$N_{a}=N_{a1}+N_{a2}+\cdots +N_{ax}+\cdots +N_{an},(1\leq x\leq n,x\in N_{+})$$

(21) $$Na=e_{a1}\times S_{a1}+e_{a2}\times S_{a2}+\cdots \frac{5x\left(2r-10x+5\right)}{6}+\cdots e_{an}\times S_{an},\left(1\leq x\leq n,x\in N_{+}\right)$$

(22) $$N=N_{a}+N_{b}+N_{c}+N_{d}+N_{e}+N_{f}^{}$$

Through comparative analysis of the compressive strength predicted by nondestructive testing and the physical compressive capacity measured by mechanical testing (Table 2), it was found that the wave-drag modulus can be used to predict the compressive capacity of scaled specimens with relatively high detection accuracy. The results demonstrate that it is suitable for the evaluation and quantitative analysis of internal defects within the timber components of ancient buildings, with an error value of δ < 15%.

Simultaneously, it was found that the central cavity or defective area in a timber column caused by sapwood cutting is inversely proportional to its compressive capacity. In other words, the larger the damaged area is, the smaller the compressive capacity. If the damage radius is the same, the higher the damage height is, the lower the compressive capacity. The part of the sapwood that is damaged has a greater impact on the compressive capacity. This is because an unbalanced damaged column is more prone to deformation or displacement under load, which causes a decrease in its compressive capacity.

### 4.5. Prediction of the Vulnerable Section

**Figure 16:**
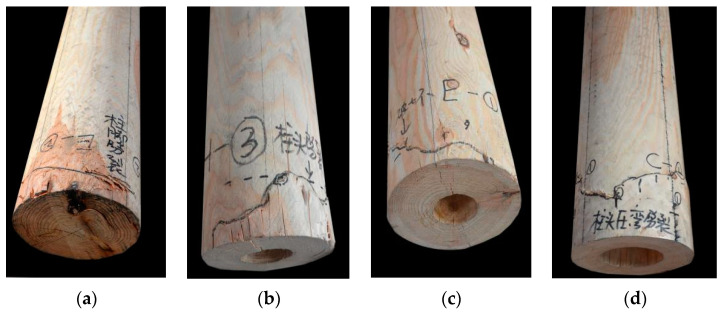
Load failure mode of central cavity: (**a**) The rising of local fibers; (**b**) Vertical cracks; (**c**) Ring cracks along the direction of the annual rings; (**d**) Wrinkes.

In ancient timber-framed buildings, wood density and damage are highly correlated to elastic modulus and mechanical strength. Compressive capacity destructive testing indicates that, owing to the different damage types, sizes, and locations, as the loading level increases, wooden components of ancient timber-framed buildings will be displaced or partially damaged, resulting in unloading. Using initial failure as the analysis benchmark, the wave-drag modulus prediction for compressive strength and failure mode in the vulnerable section is as follows:

At the initial loading stage, there is no obvious damage to the exterior of specimens A_1_–A_2_. When the bearing capacity decreases significantly, each section is squeezed but the forces are balanced. At this point, the specimen failure mode is the formation of wrinkles of the bottom outer edge material or the rising of local fibers (Figure 16a). When there is damage in the form of an internal cavity, because relatively more material is missing in the middle, as the damaged area continues to expand, the load-bearing section becomes smaller. At this point, the external fibers facing the load damage site exhibit multiple stripes in the vertical direction of the annual rings, and the bottom is loaded with ring cracks along the direction of the annual rings (Figure 16b,c). When the damaged area of the cross-section is the same, as the height of the damage increases, the fibers in the damaged part facing the load will show significant visual distortion, wrinkles, splits, and external damage. In addition, there is a marked reduction in the height of specimens (Figure 16d). According to Table 2, the damage position predicted by the wave-drag modulus is consistent with the position of the actual damaged part, which shows that comparing the wave-drag modulus values of each section can effectively locate the vulnerable sections and points within a specimen.

**Figure 17:**
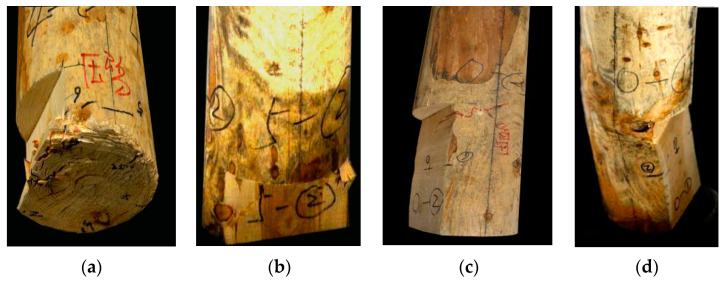
Load failure mode of sapwood reduction: (**a**) Bottom fold appears; (**b**) Local strip fracture; (**c**) Externally visible twill damage; (**d**) Serious bending deformation.

When the damaged part of the specimen is on the edge, the response to loading is as follows. When h ≤ 100 mm, in the early stage of loading, the wood component is extruded along the grain and a bottom fold appears (Figure 17a). After continuous loading, there is a local stripping of wood fiber within the specimen, accompanied by local strip fracture (Figure 17b). The surface damage of the specimen is represented as externally visible twill damage (Figure 17c). When h ≥ 300 mm, the compressive bearing capacity at the edge is degraded, and longitudinal loss and serious imbalance occur after loading, which leads to serious bending deformation at the damaged part of the specimen (Figure 17d).

According to the experiment, after loading the damaged specimen, the damage occurs in test section III. Analysis of the wave-drag modulus in the detection section (Table 2) demonstrates that the wave-drag modulus can effectively distinguish the vulnerable parts of the specimen; moreover, it verifies that the morphology of the wood component can be evaluated by nondestructive testing.

### 4.6. Prediction and Evaluation of the Residual Bearing Capacity of Timber Components

The location of damage within ancient timber-framed buildings can be determined on-site using stress wave and impedance meter nondestructive testing. This is demonstrated using the C5 timber component of the on-site ancient building as an example. Through the visualized two-dimensional stress wave graph, it can be seen that the section 200 mm away from the ground is the most severely damaged. According to the combined nondestructive testing prediction model constructed using Shapley values, it can be concluded that the internal defect area of the T section in the C5 timber component is 801.32 cm^2^. According to the wave-drag modulus prediction, the compressive capacity of this section drops by 90.64%. According to GB/T50165-2020 [28], the ratio of the material deterioration area of the bearing timber column to the area of the entire interface should be *ρ* > 1/5, which indicates that the C5 timber component has a moderate or severe impairment. In addition, the internal damage of the component results in a hollow interior; there are other damages of different sizes in the timber component in the section above 1200 mm. When the C5 timber component continues to experience loading by the upper beam, the component height of the C5 timber column will be significantly reduced. In severe cases, the timber component will present a ground layer outside, the wood fibers will be twisted and wrinkled, or the component will be slanting. In addition, the degradation of internal lignin fibers will have an impact on mechanical properties. With the degradation of compressive capacity, it is necessary to reinforce the damaged area.

## 5. Conclusions

Timber components of ancient buildings often have internal damage, which affects their bearing capacity. The wave-drag modulus of nondestructive testing was established through the analysis of 131 small wood specimens. In order to verify the effect of wave-drag modulus on residual bearing capacity, 10 scaled timber column specimens were simulated. The following conclusions were obtained:

1. Multi-probe stress wave detection can determine the location of the damaged interface. The impedance meter can determine the location of the internal damage by means of a single path, which can be used to screen the status of the internal material of timber components with large areas. Combined stress wave and impedance meter nondestructive testing can establish the internal defect combination model based on the Shapley method; this facilitates the location of the damaged area inside a timber component on-site.
2. Through the small specimen material performance experiment, it was found that there is a low correlation between the stress wave velocity and the compressive strength. Density, as an important parameter in elastic modulus, has a relatively large correlation with the impedance meter relative resistance value. Comparison of wave-drag modulus (*Fv^2^*) and measured mechanical properties indicates that there is a significant correlation between compressive strength along the grain direction and wave-drag modulus, with higher accuracy than any single method.
3. The established wave-drag modulus was verified by performing mechanical experiments on a wooden column scale model. The results show that the residual bearing capacity predicted by the model is consistent with the measured values. Simultaneously, by comparing the wave-drag modulus of different sections, the vulnerable section can be quickly located.
4. When the bottom of the wooden pillar is damaged, there are certain differences in the damage mode due to the type, size, and location of the damage. If the damaged area is small, the external visible damage is not significant, but the internal damage can be observed by stress wave and impedance meter nondestructive testing. When damage with the same area but different heights occurs in the middle cavity, the comparison of residual bearing capacity is not significant. When the damage type is a defect in the edge material of the specimen, there is a serious imbalance in the damaged part, which leads to serious visible bending deformation on the outside of the specimen and an obvious decrease in residual bearing capacity. As the damage height increases, the residual bearing capacity also decreases significantly. Thus, the nondestructive stress wave and impedance meter testing method can identify the damage type and location and provide the basis for wood component repair and reinforcement in ancient buildings.

## Figures and Tables

**Table 1:** Design parameters of reduced scale specimen.

Section Names	Height (mm)	Diameter (mm)	Type	Defective Parts	Defect Height (mm)	Defect Diameter (mm)
A_1_–A_2_	1600	200	Undamaged	-	0	0
B_1_–B_2_	1600	200	Core material cavity	Bottom	100	64
B_3_–B_4_	1600	200	Core material cavity	Bottom	300	64
C_1_–C_2_	1600	200	Edge damage	Bottom	100	160
C_3_–C_4_	1600	200	Edge damage	Bottom	300	160

**Table 2:** Comparison of measured compressive capacity and nondestructive prediction using wave-drag.

Specimen	Mechanical Test Value/kN	Nondestructive Testing Predicted Value/KN				δ/%
		Test Section-I	Test Section-II	Test Section-III	Damaged Section	
A_1_	893.071	989.36	989.364	973.353	973.353	8.99
A_2_	901.374	989.360	989.368	989.363	989.360	9.76
B_1_	705.417	970.742	954.441	635.153	635.153	9.96
B_2_	704.705	971.365	967.671	639.241	639.241	9.29
B_3_	596.740	989.369	989.360	639.113	639.113	7.10
B_4_	676.541	980.356	983.561	634.38	634.387	6.23
C_1_	616.995	923.571	975.763	568.057	568.057	7.93
C_2_	650.008	967.679	973.351	563.672	563.672	13.28
C_3_	477.374	978.643	981.387	501.321	501.321	5.02
C_4_	401.442	972.632	975.474	457.486	457.486	13.96

## Data Availability

Not applicable.
